# Cell-type specific modulation of neocortical UP and DOWN states

**DOI:** 10.3389/fncel.2015.00370

**Published:** 2015-09-24

**Authors:** Srikanth Ramaswamy, Eilif B. Muller

**Affiliations:** Blue Brain Project, Ecole Polytechnique Fédérale de LausanneGeneva, Switzerland

**Keywords:** neocortex, oscillations, acetylcholine, UP and DOWN states, layer 5 pyramidal neurons, intrinsic burst firing, carbachol, Ca^2+^ concentration

The mammalian brain is continuously active. Even during sleep and anesthesia, a rich spectrum of spontaneous oscillatory activity occurs in neocortex. A prominent example of such rhythmic activity is the emergence of so-called UP and DOWN states in neocortical networks. UP states are plateaus of neuronal depolarization lasting for hundreds of milliseconds, which are followed by sharp transitions to more hyperpolarized voltages, called DOWN states. UP and DOWN states have been observed across a range of conditions, including sleep (Massimini and Amzica, 2001), anesthesia (Steriade et al., 1993a), and quiet wakefulness (Gentet et al., 2010; see also Constantinople and Bruno, 2011). During UP states, the diversity of neocortical neurons fire action potentials (AP) spontaneously for a collective duration ranging from about 0.2 to 0.5 s (Sanchez-Vives and McCormick, 2000; Haider et al., 2006; Ruiz-Mejias et al., 2011; for reviews see Destexhe et al., 2003; Wilson, 2008). During DOWN states, the same neurons exhibit hyperpolarized membrane potentials with a near absence of AP firing, which lasts for a duration of about 0.7–1 s, depending on experimental conditions (Ruiz-Mejias et al., 2011; Sanchez-Vives, 2012).

Rhythmic fluctuations of neuronal membrane potentials into UP and DOWN states were first observed in the striatum of locally anesthetized rats through electrophysiological recordings *in vivo* (Wilson and Groves, 1981). Since then, a number of studies have characterized UP and DOWN states, mainly in anesthetized conditions or *in vitro* slice preparations, in different regions of mammalian neocortex (Steriade et al., 1993a; Sanchez-Vives and McCormick, 2000; Cossart et al., 2003; Neske et al., 2015). Several computational roles for UP and DOWN states have been proposed, such as the generation of persistent activity in cortical circuitry underlying working memory (McCormick et al., 2003), and synaptic homeostasis during sleep (Tononi and Cirelli, 2003; Delattre et al., 2015). Studies characterizing the emergence of UP and DOWN states in awake conditions have suggested a dependence on the arousal state of the animal (Constantinople and Bruno, 2011; Hromádka et al., 2013). While *in vitro* experiments have identified a role for network interactions in the generation of UP states (Sanchez-Vives and McCormick, 2000; Cossart et al., 2003; Fanselow and Connors, 2010), the neuronal and synaptic mechanisms underlying the emergence of these oscillatory states remain elusive.

In a recent paper published in *The Journal of Neuroscience*, Lőrincz and colleagues performed experiments in mouse auditory cortex *in vivo* and *in vitro* to assess the role of tonic acetylcholine (ACh) levels in the recording medium, extracellular Ca^2+^ concentations, and the activity of electrically and morphologically diverse types of neuron in generating UP and DOWN states (Lőrincz et al., 2015). Using anesthetized mice, the authors first characterized the emergence of UP and DOWN states in layer 5 pyramidal neuron types *in vivo*, which displayed both regular spiking (RS), and intrinsically burst firing (IB) patterns of AP discharge. In a vast majority of RS pyramidal neurons, UP states emerged from membrane potentials that were relatively more depolarized than DOWN states, but not at typically observed UP state levels, before a smooth wavelike transition into DOWN states. In contrast, UP states in IB pyramidal neurons always arose from relatively hyperpolarized membrane potentials, and terminated by a sharp transition back to the DOWN state. Importantly, Lőrincz et al. (2015) identified that a specific subset of IB layer 5 pyramidal neurons that fired low-frequency bursts of APs (~0.2–2 Hz) regularly led the initiation of UP states. Previous experiments in anesthetized felines and rodents have identified vital roles for cholinergic projections arising from the tegmental nuclei, and the nucleus basalis in modulating UP states in neocortical neurons (Metherate et al., 1992; Steriade et al., 1993b). Similarly, Lőrincz et al. (2015), found that scopolamine, a commonly used selective muscarinic antagonist, curtailed the length of UP and DOWN states and related AP output, demonstrating the modulatory effect of muscarinic cholinergic receptors.

To investigate the underlying cellular mechanisms of UP state generation, Lőrincz et al. (2015) used rodent neocortical slices where cholinergic transmission observed *in vivo* was pharmacologically revived *in vitro* (Steriade et al., 1993b). Specifically, the extracellular level of ACh in *in vitro* slices was restored to that measured *in vivo* (Hsieh et al., 2000) by applying a commonly used cholinergic agonist carbachol (CCH) to the recording medium. Following CCH application, Lőrincz et al. (2015) obtained extracellular local field potential (LFP) recordings from neocortical neurons, revealing the emergence of low-frequency (~2–4 Hz) UP and DOWN oscillations in layer 5.

Further analyses of extracellular recordings by Lőrincz et al. (2015) revealed that in layer 5, CCH application caused a significant increase of spontaneous AP firing in the form of tonic activity, which preceded the emergence of LFP oscillations. Neurons that generated such spontaneous firing were named “early firing” cells. In contrast, a distinct group of neurons fired brief bursts of APs, coincident with the onset of LFP oscillations, which were called putative “network drivers.” The remainder of neurons began firing APs only after LFP oscillations were initiated (Lőrincz et al., 2015). Detailed analyses revealed that previously identified “early firing” cells were actually a subset of RS layer 5 pyramidal neurons, while “network drivers” were found to be a subset of IB layer 5 pyramidal neurons. So, what sets these “early firing” cells apart from the other RS layer 5 pyramidal neurons, and “network drivers” apart from the other IB layer 5 pyramidal neurons? Intriguingly, morphological reconstructions of both “early firing” cells and “network drivers” showed that their features were consistent with those of the well characterized thick-tufted pyramidal neuron type in layer 5 of neocortex (Chagnac-Amitai et al., 1990; Markram et al., 1997; for a review see Ramaswamy and Markram, 2015). Lőrincz et al. (2015) found that the “early firing” cells responded more readily to CCH application by fast and strong depolarization leading to spontaneous AP firing, unlike the rest of the RS pyramidal neurons. Similarly, the “network driver” cells also responded promptly to CCH application with low-frequency (~0.2–2 Hz) AP burst firing, unlike the remainder of IB pyramidal neurons (Lőrincz et al., 2015). This indicates that these two pairs of pyramidal neuron populations are each distinguished by differential CCH-evoked depolarization.

In addition, the authors recorded intracellularly from an assortment of non-pyramidal neuron types in layers 5 and 6, and subdivided these recordings into three groups based on the AP firing pattern: fast-spiking, low threshold-spiking, and unclassified cells. The distributions of membrane potentials of these three neuron groups did not exhibit prominent bimodal distributions, unlike those observed in the pyramidal neurons. In contrast with pyramidal neurons, this indicates that non-pyramidal neuron types do not display pronounced rhythmic fluctuations of the membrane potential between UP and DOWN states (Lőrincz et al., 2015), consistent with other studies (Fanselow and Connors, 2010; Neske et al., 2015).

The authors further sought to explain if synaptic interactions between neocortical neurons gave rise to the low-frequency burst firing seen in “network drivers,” and pharmacologically blocked both excitatory and inhibitory synaptic transmission, while preserving CCH in the *in vitro* recording medium (Lőrincz et al., 2015). Indeed, the blockade of synaptic transmission abolished oscillatory UP/DOWN state activity as expected, and corroborated that network oscillations are principally driven by synaptic interactions between neocortical neurons. However, this manipulation also modulated individual layer 5 pyramidal neurons in three noticeable ways. First, in IB neurons identified as “network drivers,” the blockade of synaptic activity progressively shortened the duration of UP states, until only low-frequency burst firing was observed. This demonstrates that CCH-evoked depolarization induces intrinsic rhythmic bursting in these neurons. Second, in the remainder of IB neurons, and other RS neurons that were not identified as “early firing” cells, synaptic blockade gradually reduced the amplitude of rhythmic synaptic activity, and suppressed AP firing. Third, in “early firing” RS neurons, blocking excitatory synaptic activity resulted in the progressive disappearance of DOWN states, and the appearance of regular AP firing. These pharmacological manipulations demonstrate that the presence of CCH in the *in vitro* recording medium (containing ~ 2 mM extracellular Ca^2+^) selectively depolarized a subset of IB layer 5 pyramidal neurons, “network drivers” whose rhythmic burst firing coincided exactly with the onset of UP states.

An important finding of this study by Lőrincz et al. (2015) was the identification of these so called “network drivers,” a readily recognizable electrical and morphological subset of layer 5 pyramidal neurons, which fired low-frequency brief bursts of APs, were strongly depolarized by the application of CCH, and reliably drove the initiation of UP states. Furthermore, the authors categorized the diversity of neuron types in layer 5, both electrically and morphologically, and discovered that IB layer 5 pyramidal neurons exhibited a more prominent bimodal distribution of membrane potentials than other neuron types. While this study lays a foundation for identifying important roles for specific types of neurons in controlling rhythmic UP/DOWN state oscillations, several questions remain to be addressed. An important question is whether the CCH-evoked oscillations observed by Lőrincz et al. (2015) in auditory cortex slices can also be extrapolated to other cortical areas? For example, IB layer 5 pyramidal neurons have not been observed in the developing rat somatosensory cortex (Markram et al., 1997). If UP states are always initiated by the IB layer 5 pyramidal neurons most depolarized by CCH, this might preclude CCH-induced generation of UP DOWN states in such experimental preparations. Although, Lőrincz et al. (2015) report that CCH-evoked network oscillations appear to be ubiquitous across different cortical areas, the study does not provide any quantitative comparisons. Therefore, future experiments are crucial to reveal the level of ubiquity of CCH-evoked oscillations in other cortical areas. Another fundamental question to ask is if the “network driver” subset of layer 5 pyramidal neurons, as identified by Lőrincz et al. (2015), always leads the initiation of UP states in different cortical areas? Given that layer 5 pyramidal neurons exhibit a diversity of AP firing thresholds and intrinsic firing patterns in different cortical regions (van Aerde and Feldmeyer, 2015), it remains to be seen if “early firing” cells, and “network drivers” in different cortical areas and ages are always distinct subsets of RS, and IB layer 5 pyramidal neurons, respectively. Moreover, it remains to be established if the cell type-specificity of CCH-induced depolarization is preserved throughout the neocortex of diverse mammalian species. In their study, Lőrincz et al. (2015) identify “early firing” and “network driver” neuron types mainly based on their electrophysiology, and morphology. Further experiments are also required to establish the molecular identity of “early firing” and “network driver” neuron types, complementing recent studies investigating the differential activation of somatostatin, parvalbumin, or vasoactive intestinal peptide expressing non-pyramidal neuron types during UP and DOWN states in different cortical areas (Fanselow and Connors, 2010; Neske et al., 2015). Lastly, it is also critical to investigate the ubiquity of CCH-evoked depolarization of layer 5 pyramidal neurons across stages of physiological development (Zhu, 2000), to characterize when these neurons mature into “early firing” cells, and “network drivers.”

Intriguingly, despite requiring CCH for burst firing in “network drivers” and regular UP states in a traditional *in vitro* recording medium (containing ~2 mM extracellular Ca^2+^), Lőrincz et al. (2015) found that a modified recording medium that more closely mimics Ca^2+^ levels present *in vivo* (~1–1.2 mM extracellular Ca^2+^) did not result in the intrinsic low-frequency burst firing in “network drivers,” and rhythmic UP states as a consequence, but led to sporadically occurring UP states of long durations alternating with DOWN states also of long durations (Sanchez-Vives and McCormick, 2000; McCormick et al., 2003; Lőrincz et al., 2015). The most critical omission in the series of experiments presented by Lőrincz et al. (2015) is a characterization of the firing properties of IB and “network driver” neurons in the presence of CCH under reduced levels of extracellular Ca^2+^, i.e., do “network drivers” continue to exhibit intrinsic burst firing under *in vivo* levels of Ca^2+^ and ACh? Importantly, it remains to be ascertained if the CCH-evoked intrinsic burst firing of APs by “network drivers,” which led the initation of UP states, is not specific to the experimental and pharmacological conditions employed by Lőrincz et al. (2015). It is established that a wide array of modulatory mechanisms could also alter the duration of UP states in neocortical slices, such as GABA and glutamate concentrations (Mann et al., 2009; Sanchez-Vives et al., 2010), recording temperature (Bar-Yehuda and Korngreen, 2007; Reig et al., 2010), or interactions between network activity and neuronal metabolism gated by intracellular levels of adenosine triphosphate (ATP)-dependent homeostatic mechanisms (Cunningham et al., 2006). Therefore, future experiments should assess the dependence of burst firing in “network drivers” and the rhythmicity of UP states also on these factors. In conclusion, a unifying view of how extracellular ionic concentrations, GABA and glutamate levels, homeostatic mechanisms, and the cocktail of neuromodulators such as histamine, acetylcholine, noradrenaline, dopamine, and serotonin differentially impact neocortical neuron types is necessary to elucidate cell type-specific roles in the generation and modulation of UP and DOWN states. Lőrincz et al. (2015) have provided an important piece to this puzzle.

## Conflict of interest statement

The authors declare that the research was conducted in the absence of any commercial or financial relationships that could be construed as a potential conflict of interest.
